# Exploration of the Character Representation of DNA Chiral Conformations and Deformations via a Curved Surface Discrete Frenet Frame

**DOI:** 10.3390/ijms25010004

**Published:** 2023-12-19

**Authors:** Ying Wang, He Wang, Shengli Zhang, Zhiwei Yang, Xuguang Shi, Lei Zhang

**Affiliations:** 1 MOE Key Laboratory for Nonequilibrium Synthesis and Modulation of Condensed Matter, School of Physics, Xi’an Jiaotong University, Xi’an 710049, China; phd_wy@163.com (Y.W.); w1047181605@stu.xjtu.edu.cn (H.W.); zhangsl@xjtu.edu.cn (S.Z.); yzws-123@xjtu.edu.cn (Z.Y.); 2 College of Science, Beijing Forestry University, Beijing 100083, China

**Keywords:** DNA, phase theory, torsion and bending, geodesic curve line, chiral conformation

## Abstract

While undergoing structural deformation, DNA experiences changes in the interactions between its internal base pairs, presenting challenges to conventional elastic methods. To address this, we propose the Discrete Critical State (DCS) model in this paper. This model combines surface discrete frame theory with gauge theory and Landau phase transition theory to investigate DNA’s structural deformation, phase transitions, and chirality. Notably, the DCS model considers both the internal interactions within DNA and formulates an overall equation using unified physical and geometric parameters. By employing the discrete frame, we derive the evolution of physical quantities along the helical axis of DNA, including geodesic curvature, geodesic torsion, and others. Our findings indicate that B-DNA has a significantly lower free energy density compared to Z-DNA, which is in agreement with experimental observations. This research reveals that the direction of base pairs is primarily governed by the geodesic curve within the helical plane, aligning closely with the orientation of the base pairs. Moreover, the geodesic curve has a profound influence on the arrangement of base pairs at the microscopic level and effectively regulates the configuration and geometry of DNA through macroscopic-level free energy considerations.

## 1. Introduction

Understanding the mechanisms behind DNA conformational transitions and geometric deformations is of utmost importance in multidisciplinary fields. These transitions encompass the exploration of various chiral DNA geometries, stability assessment, and the arrangement behavior of double helix structures. As these structural changes occur, the corresponding biological functions also undergo modifications, particularly with regard to the interactions between internal base pairs. Efficiently studying the process of DNA conformational transitions and deformations necessitates a rigorous and quantitative representation of the DNA structure. However, directly observing the DNA’s representation from experiments remains challenging.

Scholars have extensively researched DNA conformation and structure using various experimental and theoretical approaches. For instance, the rotor magnetic bead tracking technique has proven valuable in providing critical information on structural details, particularly overall structural deformation [1]. Molecular dynamics simulations at both atomistic and coarse grain levels have offered detailed insights into the sequence-dependent statistical mechanics properties of DNA [2,3]. On the theoretical front, several models have been employed to study specific aspects of DNA behavior. The modified Zhou, Zhang, and Ou-Yang (ZZO) model, for example, has been utilized to investigate the impact of salt ions and stretching force on DNA stretching transitions [4]. Additionally, the Poland-Scheraga model and the Peyrard-Bishop-Dauxois model have been employed to explore DNA denaturation using statistical mechanics and extend the coupling between DNA distortion and denaturation [5,6,7]. The elastic rod model and its extended versions have been used to describe DNA supercoiling [8,9]. A noteworthy contribution is Wang’s free energy model, capable of distinguishing different chirality and classifying DNA structures effectively [10]. The free energy and stability of the elastic structure play vital roles in the DNA conformational transition and geometric deformation processes, often under the influence of extreme conditions [11,12,13]. Despite the progress made, certain aspects, such as local structural properties and the underlying physical laws governing the surface parameters of DNA during deformation, remain elusive using the aforementioned methods. To gain a comprehensive understanding of the influence of these factors on DNA, it becomes imperative to establish a systematic theoretical framework that delves into more comprehensive information about the local and global aspects, thereby exploring the microscopic mechanisms of DNA conformation and geometric structure deformation.

It is important to acknowledge that obtaining certain information directly from real experimental data of DNA can be challenging. However, discrete frame theory serves as a valuable and effective means to address this limitation. This theory has numerous applications in computer graphics and geometry processing, including curve fitting, shape analysis, and other related areas [14,15,16]. The integration of discrete frame theory with other theoretical frameworks and experimental methods significantly enhances our understanding of DNA behavior. This interdisciplinary approach enables precise and quantitative analyses of DNA structure and function, paving the way for advancements in various research fields that rely on accurate modelling and interpretation of DNA properties.

Characterizing the physical laws governing DNA conformational transitions and geometric deformations presents a captivating and intriguing challenge. Our ultimate objective is to develop a representation that goes beyond mere mathematical geometry and is instead directly determined based on the fundamental physical essence of biological objects. Moreover, our representation method allows for the extraction of essential physical characteristic parameters. These parameters enable us to explore the geometry and stability during the conversion of different chiral DNA configurations and to investigate the behavior of double helix structure arrangements. Beyond this, we envision that our method and characterization results can find application in addressing biological two-dimensional deformation problems, extending beyond the scope of DNA conformational transitions and geometric structure deformations. By elucidating the physical principles underlying these processes, we aim to contribute valuable insights into the broader understanding of biological systems and their dynamic transformations. Our aspiration is for this research to pave the way for broader applications and advancements in diverse multidisciplinary fields.

In this article, we present a critical state model, employing physical gauge field theory to describe the local point-to-point interactions of DNA base pairs during chiral conformational transitions in different DNA configurations. This model establishes a clear relationship between geometric properties and energy changes in DNA. Next, we introduce the Curved Surface Discrete Frenet Frame (CSDFF), an extension of the generalized curve frame, which allows for the constraint of the curve within the surface, ensuring mutual coherence between the curve and the surface. This frame is capable of describing two-dimensional surface deformation. We not only provide the discrete frame for the local area of the curve but also furnish the transfer matrix and Euler angles that describe the local curve area. Subsequently, we apply the CSDFF to periodic DNA double helix structures and employ the above information to explore the curved surface properties of DNA secondary structures. Specifically, we visualize the microscopic details of DNA double helix surface deformation using the discrete critical state (DCS) model. To validate our findings, we calculated the DNA structures of different chiral types obtained from the Protein Data Bank (https://www.pdb.org/, accessed on 8 November 2023) and constructed models that were optimized through all-atom explicit solvent molecular dynamics (MD) simulations [17,18]. Through this approach, we identify and distinguish the various types of DNA by classifying the parameters and uncovering their respective geometric and energy laws.

Overall, our research aims to shed light on the physical and mathematical parameters underlying DNA structures and their conformational transitions. By combining theoretical models and experimental data, we strive to deepen our understanding of DNA behavior and contribute to advancements in the field of biological sciences.

## 2. Model

DNA is composed of base pairs and phosphate skeletons, which combine to form a curved, double-stranded structure. This double-stranded structure is spatially considered a banded geometric surface, which bends and rotates along the axis of the DNA helix. In addition, the central axis of the geometric surface twists spatially, forming a DNA double helix structure. Such helices are usually divided into left-handed and right-handed helices, and they are generally considered to be chiral symmetric. So, we need to find an effective model that can describe this curved surface double helix structure with a chiral structure.

For a DNA molecule with N base pairs, its conformational free energy is $F=\int_{0}^{N}Fds$, where the Landau Model is introduced by Okushima and Kuratsuji [19]:

(1) $$F=F_{s}=\frac{k_{1}}{2}\frac{d{\psi^{\prime }}^{†}}{ds}\frac{d\psi^{\prime }}{ds}+\frac{k_{2}}{2}{\left|\psi^{†}\frac{d\psi^{\prime }}{ds}\right|}^{2},$$

where s is the arc unit on the DNA helical axis. The parameters $k_{1}$ and $k_{2}$ are related to isotropic elastic bending and torsional elasticity and the details are shown in Equation (3) in this section. ${\psi^{\prime }}^{†}$ is the conjugate transpose of $\psi^{\prime }$. Landau theory is considered to be the fundamental theory describing phase transitions. It introduces the concept of symmetry breaking and uses a power series expansion of the free energy density function with ordered parameters to describe the behavior of matter during phase transitions. Through the minimum value of the free energy density function, the equilibrium stability of the material structure can be determined [20,21]. And the two-component spin-wave function ψ which described the configuration of the moving frame $\left(\vec{t},\vec{b},\vec{n}\right)$ was

(2) $$\psi =\left(\begin{array}{c} \psi_{1}\\ \psi_{2} \end{array}\right)={\left(\cos\frac{\beta }{2}e^{-i\frac{\alpha +\chi }{2}},\sin\frac{\beta }{2}e^{i\frac{\alpha -\chi }{2}}\right)}^{t}.$$

**Figure 1:**
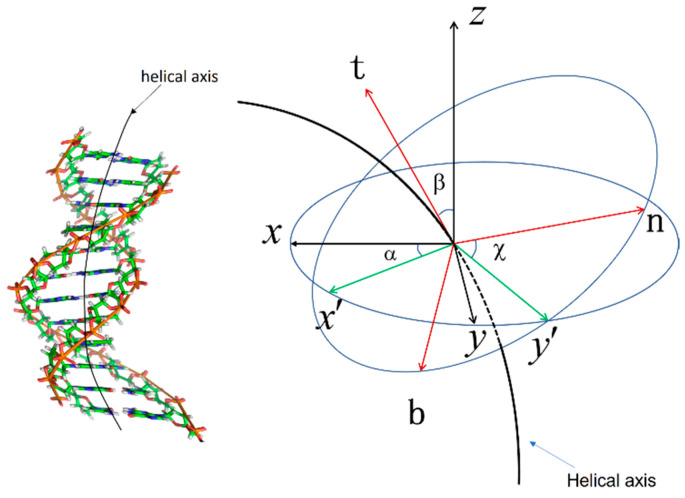
Euler angle and Euler rotation. α is the angle between the x axis and the $x^{\prime }$ axis; β is the angle between the z axis and the t axis; and χ is the angle between the $y^{\prime }$ axis and the n axis. It changes as follows. First, the rigid body is rotated counterclockwise around the z axis by angle α. Second, it is rotated around the $y^{\prime }$ axis by angle β. Finally, it is rotated around the t axis by angle χ.

This is parameterized by Euler angles [22]. The Euler angle is shown in Figure 1.

Structurally, we consider the configuration transition from B-DNA to Z-DNA, i.e., a chiral symmetry structural change driven by the gauge field ρ from the considered gauge transformation $\psi^{\prime }=\psi \left(ia\left(s\right)\right)$ [10]. For a ladder-like DNA molecule that is not affected by external forces, ρ>0 or ρ<0 if it is a right- or left-handed double helix, respectively.

By substituting the spinor $\psi^{\prime }$ into Formula (1), the free energy satisfying the gauge transformation that is obtained is as follows:

(3) $$F_{s}=\frac{B}{2}\left[{\left(\frac{d\beta }{ds}\right)}^{2}+{\left(\frac{d\alpha }{ds}\right)}^{2}\sin^{2}\beta \right]+\frac{C}{2}{\left(\frac{d\alpha }{ds}\cos\beta +\frac{d\chi }{ds}-\rho \right)}^{2},$$

where $B=\frac{k_{1}}{4}$ is the isotropic elastic bending and $C=\frac{\left(k_{1}\;+\;k_{2}\right)}{4}$ is the torsional elasticity. In Equation (3), $\frac{d\beta }{ds}$, $\frac{d\alpha }{ds}$, and $\frac{d\chi }{ds}$ represent spatial bending, spatial rotation, and cross-sectional deviation trend of the DNA helix axis, respectively. The chiral gauge potential *ρ* added to this formula ensures free energy gauge invariance and describes the non-local variation in the twist angle of the DNA helical axis due to the application of a negative torque to the DNA molecule. It is the rotational angle between consecutive base pairs and represents the interaction between the inside of the base pairs.

As the double-stranded structure of the DNA double helix molecule is regarded as a banded geometric surface [10] and differential geometry is used to describe it [23], the free energy can be related to the geometric structure of DNA as follows:

(4) $$F_{s}=\frac{B}{2}\left[k_{n}{}^{2}+k_{g}{}^{2}\right]+\frac{C}{2}{\left(\tau_{g}+\frac{d\chi }{ds}-\rho \right)}^{2},$$

where

(5) $$\left\{\begin{array}{c} k_{g}=-\sin\beta \frac{d\alpha }{ds}\\ k_{n}=\frac{d\beta }{ds}\\ \tau_{g}=\cos\beta \frac{d\alpha }{ds} \end{array}\right.,$$

where $k_{n}$ is the normal curvature of the helical axis, $k_{g}$ is the geodesic curvature of the helical axis, and $\tau_{g}$ is the geodesic torsion of the helical axis. The parameter $k=\sqrt{k_{n}{}^{2}+k_{g}{}^{2}}$ is the overall curvature of the surface, and $\tau =\sqrt{{\left(\tau_{g}+\frac{d\chi }{ds}-\rho \right)}^{2}}$ is the overall torsion of the surface.

For the steady-state DNA configuration free energy, the additional free energy cost term due to changing the base pair structure along s, the effective potential should also be taken into account [19], so the total free energy is

(6) $$F=\frac{B}{2}\left(k_{n}^{2}+k_{g}^{2}\right)+\frac{C}{2}{\left(\tau_{g}+\frac{d\chi }{ds}-\rho \right)}^{2}+\frac{d_{1}}{2}{\left(\frac{d\rho }{ds}\right)}^{2}+\tau_{c}\rho .$$

Equation (6) is called the critical state model. This model describes different DNA in chiral conformational transitions by including the gauge potential and extends Landau’s theory of phase transitions. Moreover, the critical state model also clearly reflects the relationship between the geometric properties and energy of DNA; it correlates the structure and function of biomolecules and explains the possible behavior and direction of biological reactions.

## 3. Frenet Frame of Curved Surface

We start with the description of a Frenet frame in surface theory. For the generalized Frenet frame, we generally use it to describe space curves. In the classical differential geometry curve theory section, it can well describe the geometric properties of one-dimensional curves such as bending and twisting. However, in the curved surface, the curve C falls on the surface S, and the generalized Frenet frame and its motion formula will not reflect the mutual constraint relationship between the curve C and the surface S. Therefore, we establish a curved surface continuous frame, taking the DNA double helix structure as an example, which is suitable for all curved surface torsion research.

### 3.1. The Continuous Frenet–Serret Frame of Curved Surface

For DNA, because of its special curved surface structure formed by double strand winding, we need to study the helical axis at the center of the double-strand as a curve in a curved surface to describe the structure of the DNA double helix. An orthogonal frame field along the curve C, denoted as $\left(\vec{r}(s),\vec{t},\vec{b},\vec{n}\right)$, is used to consider both the curve C and the surface S.

The Frenet–Serret Frame is defined as follows [10,24].

The coordinates satisfy the following equation:

(7) $$\frac{d}{ds}\left(\begin{array}{c} \vec{t}\\ \vec{b}\\ \vec{n} \end{array}\right)=\left(\begin{array}{ccc} 0 & k_{g} & k_{n}\\ -k_{g} & 0 & \tau_{g}\\ -k_{n} & -\tau_{g} & 0 \end{array}\right)\left(\begin{array}{c} \vec{t}\\ \vec{b}\\ \vec{n} \end{array}\right),$$

where

(8) $$\left\{\begin{array}{c} k_{g}=-\sin\beta \frac{d\alpha }{ds}\\ k_{n}=\frac{d\beta }{ds}\\ \tau_{g}=\cos\beta \frac{d\alpha }{ds} \end{array}\right.,$$

(9) $$k=\sqrt{k_{g}^{2}+k_{n}^{2}}=\sqrt{{\left(\frac{d\beta }{ds}\right)}^{2}+\sin^{2}\beta {\left(\frac{d\alpha }{ds}\right)}^{2}}.$$

For the DNA double helix in a double strand curved surface, the $\vec{r}\left(s\right)$ is the parametric curve of the helical axis, and $\vec{t}$ is the unit tangent vector at a point of the helical axis:

(10) $$\vec{t}=\frac{d\vec{r}\left(s\right)}{ds}.$$

Rotate the unit tangent vector counterclockwise by $\frac{\pi }{2}$ to obtain the in-plane normal vector:

(11) $$\vec{n}=\left(\cos\beta \cos\alpha ,\cos\beta \sin\alpha ,-sin\beta \right).$$

Then the third unit vector is

(12) $$\vec{b}=\vec{n}\times \vec{t}=\left(\sin\alpha ,-\cos\alpha ,0\right).$$

**Figure 2:**
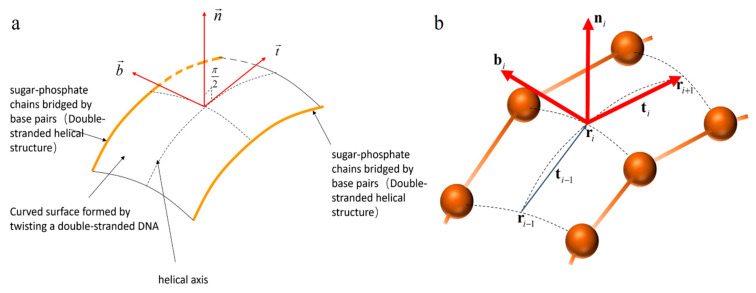
(**a**) The continuous frame on the DNA curved surface. (**b**) The discrete frame on the DNA curved surface. The orange balls represent nucleotide positions on the corresponding chain.

The frame $\left(\vec{r}(s),\vec{t},\vec{b},\vec{n}\right)$ described the Frenet frame of the helical axis falling on the DNA surface; see Figure 2. $k_{g},k_{n},\tau_{g}$ described the degree of curvature and twist of the curved surface.

### 3.2. The Discrete Frenet–Serret Frame of Curved Surface (CSDFF)

For a two-dimensional surface S constrained by two curves $l_{i},l_{i}{}^{\prime }$, we use a recursively defined frame $\left\{D_{i}\right\}=\left\{\left(n_{i},b_{i},t_{i}\right)\right\}$ to describe the position of the centerline C on the surface S. The reason for this is that the DNA helical axis is located on a two-dimensional curved surface composed of double nucleic acid strands. We need to use the helical axis as the position parameter to describe the specific information of each site, and the helical axis is just the center of the two chains. Therefore, we take the midpoint of the two chains as the position parameter $r_{i}$ of the spiral axis, and use $r_{i}$ to trace the position of the spiral axis C curve at point i.

The helix axis parameter $r_{i}$ is at the center of the two nucleotide chains $l_{i},l_{i}{}^{\prime }$; we define the position vector:

(13) $$r_{i}=\frac{1}{2}\left(l_{i}+l_{i}{}^{\prime }\right),$$

wherein are the position parameters of chain $l_{i}$ and chain $l_{i}{}^{\prime }$ respectively, wherein the symmetrical deoxynucleotides on the two chains at the i site are connected according to the principle of complementary base pairing.

Next, we construct the Frenet frame at point i by using the position information at points i−1 and i+1 of the spiral axis curve. The tangent vector of the curve at i point is

(14) $$t_{i}=\frac{r_{i+1}-r_{i}}{\left|r_{i+1}-r_{i}\right|}.$$

The second vector consists of the parameters of the tangent vector at i−1 and i:

(15) $$b_{i}=\frac{t_{i-1}\times t_{i}}{\left|t_{i-1}\times t_{i}\right|}\left(t_{i-1}\times t_{i}\neq 0\right).$$

The in-plane normal vector is

(16) $$n_{i}=\frac{b_{i}\times t_{i}}{\left|b_{i}\times t_{i}\right|}.$$

The frame $\left(n_{i},b_{i},t_{i}\right)$ forms a discrete frame for the DNA curved surface shown in Figure 2b. The frame gives an ordered collection of all the positions on the DNA helical axis.

The transfer rule of the Frenet frame at i+1 and i is

(17) $$\left(\begin{array}{c} n_{i+1}\\ b_{i+1}\\ t_{i+1} \end{array}\right)=R_{i+1,1}\left(\begin{array}{c} n_{1}\\ b_{1}\\ t_{1} \end{array}\right),$$

which $R_{i+1,1}=R_{i+1,i}\cdot R_{i,i-1}\cdot \ldots \cdot R_{3,2}\cdot R_{2,1}$. Every Euler rotation here is relative to the rotation of the base.

We choose the $\left(zyz\right)$ angles, and the transfer matrix is

(18) $$R_{i+1,1}=\left(\begin{array}{ccc} \cos\alpha \sin\beta \cos\gamma -\sin\alpha \sin\gamma & -\cos\alpha \cos\beta \sin\gamma -\sin\alpha \cos\gamma & \cos\alpha \sin\beta \\ \sin\alpha \cos\beta \cos\gamma +\cos\alpha \sin\gamma & -\sin\alpha \cos\beta \sin\gamma +\cos\alpha \cos\gamma & \sin\alpha \sin\beta \\ -\sin\beta \cos\gamma & \sin\beta \sin\gamma & \cos\beta \end{array}\right).$$

The angle α, β and χ represents the Euler rotation angle of point i+1 relative to point 1.

We set the transfer matrix as

(19) $$R_{i+1,1}=\left(\begin{array}{ccc} r_{11} & r_{12} & r_{13}\\ r_{21} & r_{22} & r_{23}\\ r_{31} & r_{32} & r_{33} \end{array}\right).$$

Then the Euler angle at i+1 is expressed as

(20) $$\left\{\begin{array}{c} \alpha_{i}=\arctan2\left(\begin{array}{cc} \frac{r_{23}}{\sqrt{r_{13}^{2}+r_{23}^{2}}}, & \frac{r_{13}}{\sqrt{r_{13}^{2}+r_{23}^{2}}} \end{array}\right)\\ \beta_{i}=\arctan2\left(\begin{array}{cc} \sqrt{r_{13}^{2}+r_{23}^{2}}, & r_{33} \end{array}\right)\\ \gamma_{i}=\arctan2\left(\begin{array}{cc} \frac{r_{32}}{\sqrt{r_{13}^{2}+r_{23}^{2}}}, & -\frac{r_{31}}{\sqrt{r_{13}^{2}+r_{23}^{2}}} \end{array}\right) \end{array}\right..$$

In this way, we can get the Euler angle of each site by the Frenet frame of each site. $\left\{D_{i}\right\}=\left\{\left(n_{i},b_{i},t_{i}\right)\right\}$ and $\left(\alpha_{i},\beta_{i},\gamma_{i}\right)$ give a representation to describe the frame at any position on the helical axis of the DNA.

### 3.3. Periodic Euler Angles

For periodic DNA helices, the Euler angles on the helical axis are also periodic. Therefore, when calculating the Euler angle difference between each site and adjacent sites, its periodicity needs to be taken into consideration. We do the following processing:

1. We set that the value range of the Euler angle is 0 to 2π. When using the 2-argument arctangent (arctan2) function to calculate the Euler angle, the value range of this function is −π to π, which cannot show the correct Euler rotation direction. So, we need to place the value range of the result in 0 to 2π by determining the tangent value of the corresponding position.

The 2-argument arctangent function is

(21) $$\theta =\arctan2\left(\begin{array}{cc} \sin\theta , & \cos\theta \end{array}\right).$$

When

(22) sinθ<0,

Then

(23) θ→θ+2π;

2. We calculate the Euler angle difference of adjacent sites by adding the period. In the calculation of the Euler angle difference near the edge of the cycle, we calculate the Euler angle difference between adjacent points by adding a cycle 2*π* to the latter point to ensure the correctness of the Euler angle rotation direction:

(24) $$\Delta \theta =\theta_{i+1}+2\pi -\theta_{i}.$$

## 4. DCS Model and Test DNA

As a verification, we use the DCS model to study the different chiral structures of DNAs, especially B-DNA and Z-DNA structures.

### 4.1. DCS Model

To measure the actual free energy of the DNA, we discretize Formula (6). By discretizing the critical state model, the free energy density at point i of DNA is expressed as

(25) $$F_{i}=\frac{B_{i}}{2}\left(k_{n_{i}}{}^{2}+k_{g_{i}}{}^{2}\right)+\frac{C_{i}}{2}{\left(\tau_{g_{i}}+\frac{\Delta \chi_{i+1,i}}{\Delta s_{i,i-1}}-\Delta \rho_{i+1,i}\right)}^{2}+\frac{d_{1}}{2}{\left(\frac{\Delta \rho_{i+1,i}}{\Delta s_{i,i-1}}\right)}^{2}+\tau_{c}\Delta \rho_{i+1,i},$$

where geodesic curvature, normal curvature, and geodesic torsion at the point i are

(26) $$\left\{\begin{array}{l} k_{n_{i}}=\frac{\Delta \beta_{i+1,i}}{\Delta s_{i,i-1}}\\ k_{g_{i}}=-\frac{\Delta \alpha_{i+1,i}}{\Delta s_{i,i-1}}\sin\beta_{i}\\ \tau_{g_{i}}=\frac{\Delta \alpha_{i+1,i}}{\Delta s_{i,i-1}}\cos\beta_{i} \end{array}\right.,$$

which $\Delta \alpha_{i+1,i}=\alpha_{i+1}-\alpha_{i}$, $\Delta \beta_{i+1,i}=\beta_{i+1}-\beta_{i}$, $\Delta \chi_{i+1,i}=\chi_{i+1}-\chi_{i}$. It should be noted here that the frame $\left\{D_{i}\right\}=\left\{\left(n_{i},b_{i},t_{i}\right)\right\}$ is obtained by the information through the site i+1 and i−1 points. The value of the Euler angle is obtained through the information of i−1 and i−2. Therefore, when calculating the difference between the Euler angle, the information of the total four points of the head and tail is a blind spot. The parameter $\Delta s_{i,i-1}=\left|{\vec{r}}_{i}-{\vec{r}}_{i-1}\right|$ is the distance between adjacent base pairs, because each site is set on the average location of each base plane, and the minimum unit is 1 bp. Here we set Δρ=Δχ, because ρ is the rotation angle between consecutive base pairs in the helix.

The isotropic bending elasticity B and torsional elasticity C is

(27) $$B_{i}=\frac{b}{\Delta s_{i,i-1}}K_{B}T_{R}=\frac{b}{\Delta s_{i,i-1}\times 10^{-1}}\times 4.1\times 10^{-21}\;J,$$

(28) $$C_{i}=\frac{c}{s_{i,i-1}}K_{B}T_{R}=\frac{c}{\Delta s_{i,i-1}\times 10^{-1}}\times 4.1\times 10^{-21}\;J.$$

with parameter values $d_{1}=D_{1}/\omega_{0}{}^{2}$, where $D_{1}=4.1\times 10^{-21}\;\;J$ and $\omega_{0}=0.6\;\mathrm{rad}/\mathrm{pb}$ [19]. The critical torque is $\tau_{c}=-7.9\times 10^{-21}\;J$ [25]. Bending persistence length b and Twisting persistence length c refer to the literature [26].

### 4.2. Parameter Acquisition and Calculation

We obtain the three-dimensional coordinates of all the different atoms in DNA, including two types of DNA, B-DNA and Z-DNA, respectively. The structure of B-DNA was obtained from the PDB database. The PDB ids used in this article are shown in Supplementary Table S1 [27,28,29,30,31,32,33]. Because of missing Z-DNA experimental structures with sufficient length (containing at least one helical period), the structure of Z-DNA was conducted by the homology model method (Accelrys. Discovery Studio 3.1. Available online: http://accelrys.com (accessed on 8 November 2022)) and refined by the 200-ns explicit solvent molecular dynamics (MD) simulations using GROMACS 2018.8 [34] and Charmm36m force field [35]. Details of the simulation were published previously [17,18,36] and can be found in Supplementary Information (Figure S6).

The geometric structures such as overall bending and twisting are described by the central helical axis of the two DNA phosphate backbones $l_{i}$ and $l_{i}{}^{\prime }$. When performing backbone selection for both chains, a good set of positions needs to be selected to properly describe the DNA helical structure. What we use here is to select the average position of atoms $C_{3}{}^{\prime },C_{4}{}^{\prime },O_{3}{}^{\prime }$ and $C_{1}{}^{\prime }$ of each deoxynucleotide to represent the position on the backbone chain $l_{i}$:

(29) $$l_{i}=\frac{C_{3}{}^{\prime }+C_{4}{}^{\prime }+O_{3}{}^{\prime }+C_{1}{}^{\prime }}{4},$$

The selection of another position on the chain $l_{i}{}^{\prime }$ according to the principle of complementary base pairing is also the above method. Of course, the method of definition is not the only one. The reason for our choice is that deoxyribose is the intermediate unit linking the base and the phosphate backbone, and taking the average of atoms can better represent the position of the deoxyribose.

We modelled the helical axis by treating $r_{i}$ as vertices in a discrete piecewise curve, and used it and the transfer matrix to characterize the frame and Euler angles describing any position along the DNA helical axis. We used the Python program to parse the DNA coordinates to give the frame and Euler angles along the helical axis. Then we calculated the corresponding parameters such as the free energy density of the DNA and the physical properties of its chiral configuration through Formula (25).

## 5. Results and Discussion

This section uses the DCS model, which combines surface discrete frame theory with gauge theory and Landau phase transition theory, to identify and distinguish DNA with different chiral structures, and uses the difference in free energy and physical properties as characterizations to explore the microscopic properties and macroscopic mechanisms during DNA structure changes.

Through the discrete frame approach, we have derived the evolution of various physical quantities along the helical axis of DNA, such as free energy density, gauge potential, geodesic curvature, geodesic torsion, and more. Other parameters besides those in this section are listed in Supplementary Information.

### 5.1. Free Energy and Gauge Potential

**Figure 3:**
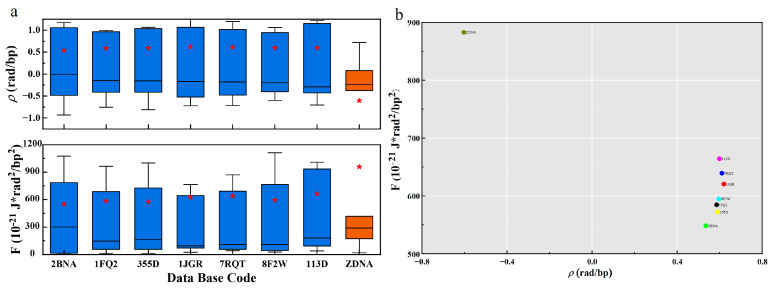
The energy distribution of different DNA structures. (**a**). Boxplots of F and ρ for different PDBs. The blue data indicate the B-type structure, the orange data indicate the Z-type structure, and the red stars indicate the average values. (**b**). PDB distribution plot of mean F and mean ρ.

The gauge potential of B-DNA is positive, and the distribution of gauge potential and PDB in Figure 3 shows that it is mainly distributed around 0.6 rad. The gauge potential of Z-DNA is negative and its value is mainly around −0.6 rad. This agrees with the results in the literature [19,37]. The mean values of the gauge potentials of the two structures are symmetrically distributed. From the Section 2, we know that the gauge potential ρ describes the rotation angle between consecutive base pairs, and represents the interaction between the base pairs. When the gauge potential is positive, adjacent base pairs rotate counterclockwise and stack sequentially to form a right-handed helix, so the base pair plane shows a negative twisting tendency along the helical axis to form B-DNA. Similarly, when the gauge potential is negative, the adjacent base pairs rotate clockwise and arrange in turn as a left-handed helix, so the base pair plane shows a positive twisting tendency along the helical axis to form Z-DNA. Since the absolute values of the gauge potentials are almost the same, the two structures are chirally symmetric.

At the same time, we know from the Section 2 that the torsion is composed of geodesic torsion, initial angle change rate, and gauge potential. Therefore, the interaction between base pairs not only affects the way adjacent bases are arranged, but also contributes to the twisted part. In addition, it also contributes to the effective potential (Supplementary Figure S2).

The free energy and PDB distribution plots in Figure 3 show that B-DNA and Z-DNA have significant differences in energy. The free energy density of B-DNA with a right-handed helical structure is significantly lower than that of Z-DNA with a left-handed helical structure, and the B-DNA is concentrated around 600, while the Z-DNA is distributed around 970. This result well demonstrates the energetic and structural characteristics of the two DNA secondary structures with chiral symmetry, and the free energy results indicate that B-DNA is more structurally stable than Z-DNA. This is consistent with the fact that DNA usually exists in the form of B-DNA with lower energy density in biologically active cells. Only in biological processes such as transcription [38] may some Z-DNA with higher energy density be generated. In addition to the overall free energy, we also present the relationship between the total free energy density F and the global geometric deformation-related free energy Fs in Supplementary Figure S1. Supplementary Figure S1 and Supplementary Table S1 show that Fs contributes a large and major part of the overall free energy.

### 5.2. Curvature and Torsion

**Figure 4:**
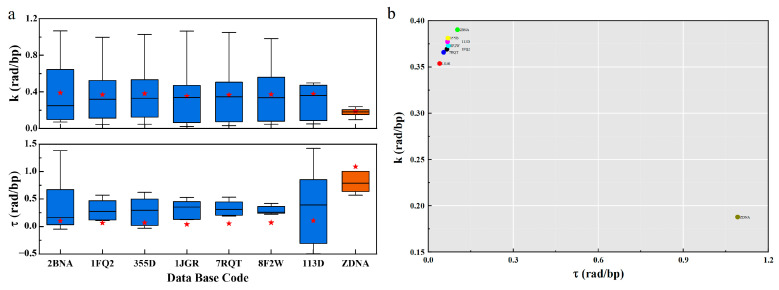
The geometric distribution of different DNA structures. (**a**). Boxplots of k and τ for different PDBs. The blue data indicate the B-type structure, the orange data indicate the Z-type structure, and the red stars indicate the average values. (**b**). The PDB distribution map of k and τ.

The PDB distribution of curvature and torsion in Figure 4 shows a significant difference in geometric properties between B-DNA and Z-DNA. The curvature of B-DNA is slightly higher than that of Z-DNA, with B-DNA concentrated around 0.4 and Z-DNA distributed around 0.21; In the torsion section, Z-DNA has a greater torsion than B-DNA. The values of B-DNA are mainly concentrated around 0.07, while the Z-DNA structure is at 1.1, and their mean values are distributed symmetrically.

This seems to imply that when the configuration of DNA changes, for B-DNA, there is always a tendency to change the curvature, while the geodesic torsion remains relatively stable (Supplementary Figure S3). This property makes DNA more prone to bending, forming supercoiled structures. At the same time, it keeps the free energy density low for stability. Based on this, B-DNA can easily wrap around octamers on a large scale to form nucleosomes, further form chromatin, and then be compressed and assembled into a small nucleus.

The greater torsion of Z-DNA seems to indicate that Z-DNA is less likely to be bent to form a supercoiled structure. Z-DNA is more inclined to form geometric configurations with more torsion. Forcibly increasing the curvature of Z-DNA may lead to the instability of Z-DNA, and then lead to the damage of Z-DNA geometric configuration. The larger torsion leads to the higher free energy density of Z-DNA. This also seems to imply that in the formation of the geometric configuration of DNA, the contribution of torsion to free energy is greater, while the curvature is relatively small.

DNA has chirality, and the natural right-handed state is the best choice. DNA in this state more easily maintains stability and has greater redundancy in curvature and torsion. However, Z-DNA is an “unnatural” state. Larger curvature may destroy its geometric structure, and there is a small redundancy in curvature and torsion. Therefore, it can be speculated that there is an order in the change of the geometric configuration of DNA, firstly the curvature, then the torsion. Second, the change of curvature and torsion of Z-DNA has a small redundancy, which will only occur under certain conditions.

In addition, as shown in Figure 3 and Figure 4, there are great differences in free energy and geometric parameters between the right-handed conformation B-DNA in the blue part and the left-hand conformation Z-DNA in the orange part. The main reason may be that the interactions between base pairs are different. Specifically, we believe that the interactions determine the chiral conformation and geometric arrangement.

### 5.3. Geodesic Curvature and Normal Curvature

**Figure 5:**
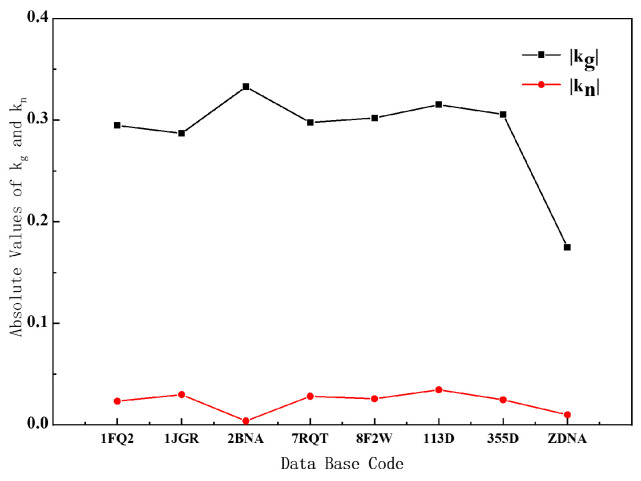
Absolute value distributions of geodesic and normal curvatures of different DNA structures.

We decompose the curvature into geodesic curvature and normal curvature, and we get some important conclusions by analyzing the relevant data of B- and Z-DNA. It can be seen from the curve in Figure 5 that whether it is B-DNA or Z-DNA, the absolute value of their geodesic curvature is much larger than the normal curvature. Combining Equations (8) and (9) and Supplementary Figures S4 and S5, the contribution of geodesic curvature to the overall curvature is the main factor, and it can basically represent the change in the overall curvature. The reason for this result is that the Euler angle β varies very little with the arc element, so the normal curvature is very small. But α varies greatly with the arc element, so the geodesic curvature is relatively large.

Understand it from the point of view of differential geometry. For B-DNA and Z-DNA, we can think that the helical axis is just on the helical surface, that is to say, the helical axis is a curved surface curve, so the geometric shape of the helical axis must be restricted by the curved surface. In a helical plane, the helical axis and the base-pair plane are nearly perpendicular. Our analysis indicates that the geodesic curvature of the helical axes of B- and Z-DNA is much larger than the normal curvature. This fact shows that the direction of the base pairs of B- and Z-DNA on the helical plane is closer to the geodesic curve of the helical plane, which is crucial for the arrangement of the base pairs. Because of the property that the shortest distance between two points on a surface must be along the geodesic curve, in DNA, only the base pairs along the geodesic curve can ensure that the distance between the base pairs is the shortest and their arrangement is the closest.

In addition, we calculated the free energy density function of DNA according to the curvature and torsion of the helical axis. In fact, this is also a calculation of the geodesic-related free energy density function on the DNA helical surface. In general, we can define the Lagrange density function on the geodesic curve, and the action calculated with the Lagrange density function should be kept to a minimum. This also seems to indicate that the orientation of the base pair in the helical plane close to the helical plane geodesic curve is one of the conditions for DNA to maintain its lowest state of free energy density function. This is important for maintaining a stable state of DNA.

From the perspective of base pair interaction, for geodesic curvature, it belongs to the quantity of intrinsic geometry of the surface, and it is related to the essential reason of double helix arrangement. In more detail, the change of Euler angle α with the arc element can describe the angular twist caused by the steric hindrance effect of the chemical structures of purine and pyrimidine and deoxyribose, which are not in the same plane. The torsion of this angle can be expressed by geodesic curvature and geodesic torsion. In other words, the deformation in the base plane is mainly expressed by geodesic curvature and geodesic torsion. For the interactions between adjacent base planes, we describe them by gauge potentials. Geodesic curvature, geodesic torsion, and gauge potential together describe the DNA double helix arrangement of different chiral types.

## 6. Conclusions

In this study, the DCS model was proposed, innovatively combining surface discrete frame theory with gauge theory and Landau phase transition theory. The aim was to investigate the structural deformation of DNA and explore its phase transitions and chirality. Compared to other methods, our model not only took into account the internal interactions within DNA but also formulated an overall equation using unified physical or geometric parameters. This approach struck a balance between studying DNA transformations at the molecular level and providing a uniform description of global conformations, yielding more straightforward insights into the underlying physical meanings.

By employing the discrete frame approach, we derived the evolution of various physical quantities along the helical axis of DNA, including free energy density, gauge potential, geodesic curvature, geodesic torsion, and more. Our results revealed that the gauge potential played a pivotal role in determining the arrangement and chirality of the double helix structure. Specifically, the B-DNA with positive gauge potential exhibited lower free energy compared to the Z-DNA with negative gauge potential. This outcome was attributed to the significant impact of local chiral structure changes on the overall DNA structure’s torsion, and the curvature and torsion of the overall geometry significantly contributed to the global free energy. Consequently, B-DNA, with its right-hand structure, proves more stable than the left-handed Z-DNA.

Furthermore, our findings shed light on the geometric properties of DNA with different chiral structures. B-DNA displayed larger curvature, facilitating bending and coiling during nucleosome formation, enabling the assembly of large-scale DNA into compact bodies in the nucleus. On the other hand, Z-DNA, an unusual form produced during transcription [38], possessed smaller curvature, enhancing shape retention during transcription, effectively maintaining a straight-line conformation.

Additionally, we discovered that the direction of DNA base pairs on the helical plane closely aligned with the geodesic curve of the helical plane. This alignment allowed DNA to maintain the lowest free energy density function, crucial for the stability of the DNA double helix structure.

By exploring the law of DNA double helix structure formation and its influencing factors, we identified that deformation in the base plane primarily depended on geodesic curvature and geodesic torsion, while the interaction between adjacent surfaces was primarily influenced by the gauge potential. These three parameters effectively described the arrangement of DNA double helices with different chiral types.

In conclusion, this paper, based on the DCS model, provided an in-depth exploration of the microstructure characteristics and macroscopic deformation mechanism during the DNA structure change process. The results demonstrated the model’s effectiveness in studying the deformation of two-dimensional surfaces and conformational transitions. Moreover, we anticipate that with enough structural samples, our method could be extended to analyze arbitrarily long 2D deformable bodies, facilitating the study of their local and global biophysical characteristics during dynamic configuration transformations in the future.

## Data Availability

The data presented in this study are available on request from the corresponding author.

## Supplementary Materials

The supporting information can be downloaded at: https://www.mdpi.com/article/10.3390/ijms25010004/s1. References [27,28,29,30,31,32,33,34,35,36,39,40,41,42,43] are cited in the supplementary materials.

## References

[1] Lebel P., Basu A., Oberstrass F.C., Tretter E.M., Bryant Z. (2014). Gold rotor bead tracking for high-speed measurements of DNA twist, torque and extension. Nat. Methods.

[2] Skoruppa E., Voorspoels A., Vreede J., Carlon E. (2021). Length-scale-dependent elasticity in DNA from coarse-grained and all-atom models. Phys. Rev. E.

[3] Wu Y.Y., Bao L., Zhang X., Tan Z.J. (2015). Flexibility of short DNA helices with finite-length effect: From base pairs to tens of base pairs. J. Chem. Phys..

[4] Dong R.X., Yan X.L., Yu G.F., Liu S.G. (2003). B- to S-form transition of double-stranded DNA in solutions of various salt concentrations. Phys. Lett. A.

[5] Poland D., Scheraga H.A. (1966). Phase transitions in one dimension and the helix-coil transition in polyamino acids. J. Chem. Phys..

[6] Peyrard M., Bishop A.R. (1989). Statistical mechanics of a nonlinear model for DNA denaturation. Phys. Rev. Lett..

[7] Dauxois T., Peyrard M., Bishop A.R. (1993). Dynamics and thermodynamics of a nonlinear model for DNA denaturation. Phys. Rev. E.

[8] Daniels B.C., Forth S., Sheinin M.Y., Wang M.D., Sethna J.P. (2009). Discontinuities at the DNA supercoiling transition. Phys. Rev. E.

[9] Liverpool T.B., Harris S.A., Laughton C.A. (2008). Supercoiling and denaturation of DNA loops. Phys. Rev. Lett..

[10] Wang Y., Shi X.G. (2018). Knot soliton in DNA and geometric structure of its free-energy density. J. Biol. Phys..

[11] Liu M.Q., Cui Y.X., Zhang Y.P., An R., Li L., Park S., Sugiyama H., Liang X.G. (2022). Single base-modification reports and locates Z-DNA conformation on a Z-B-chimera formed by topological constraint. Bull. Chem. Soc. Jpn..

[12] Oberstrass F.C., Fernandes L.E., Bryant Z. (2012). Torque measurements reveal sequence-specific cooperative transitions in supercoiled DNA. P. Natl. Acad. Sci. USA.

[13] Bryant Z., Stone M.D., Gore J., Smith S.B., Cozzarelli N.R., Bustamante C. (2003). Structural transitions and elasticity from torque measurements on DNA. Nature.

[14] Hu S., Lundgren M., Niemi A.J. (2011). Discrete Frenet frame, inflection point solitons, and curve visualization with applications to folded proteins. Phys. Rev. E Stat. Nonlin Soft Matter Phys..

[15] Botti L. (2012). Influence of Reference-to-physical frame mappings on approximation properties of discontinuous piecewise polynomial spaces. J. Sci. Comput..

[16] Tsuchie S. (2021). Reconstruction of intersecting surface models from scanned data for styling design. Eng. Comput..

[17] Perez A., Luque F.J., Orozco M. (2012). Frontiers in molecular dynamics simulations of DNA. Acc. Chem. Res..

[18] Yoo J., Winogradoff D., Aksimentiev A. (2020). Molecular dynamics simulations of DNA-DNA and DNA-protein interactions. Curr. Opin. Struct. Biol..

[19] Okushima T., Kuratsuji H. (2011). DNA as a one-dimensional chiral material: Application to the structural transition between B form and Z form. Phys. Rev. E.

[20] Landau L.D., Lifshits E.M., Pitaevskiĭ L.P. (1980). Statistical Physics.

[21] Landau L.D., Lifshits E.M., Kosevich A.d.M., Pitaevskii L.P., Lifshitz E.M., Kosevich A.M., Pitaevskii L.P. (1986). Theory of Elasticity.

[22] Sakurai J. (2014). Modern Quantum Mechanics.

[23] Chern S.-S., Chen W., Lam K.S. (1999). Lectures on Differential Geometry.

[24] Chern W.H. (1990). Differential Geometry.

[25] Marko J.F. (2007). Torque and dynamics of linking number relaxation in stretched supercoiled DNA. Phys. Rev. E.

[26] Efremov A.K., Yan J. (2018). Transfer-matrix calculations of the effects of tension and torque constraints on DNA-protein interactions. Nucleic Acids Res..

[27] Sines C.C., McFail-Isom L., Howerton S.B., VanDerveer D., Williams L.D. (2000). Cations mediate B-DNA conformational heterogeneity. J. Am. Chem. Soc..

[28] Howerton S.B., Sines C.C., VanDerveer D., Williams L.D. (2001). Locating monovalent cations in the grooves of B-DNA. Biochemistry.

[29] Drew H.R., Samson S., Dickerson R.E. (1982). Structure of a B-DNA dodecamer at 16-K. Proc. Natl. Acad. Sci. USA.

[30] Chen C., Fang Z., Huang Z. (2022). 2’-beta-selenium atom on thymidine to control beta-form DNA conformation and large crystal formation. Cryst. Growth Des..

[31] Ogbonna E.N., Paul A., Farahat A.A., Terrell J.R., Mineva E., Ogbonna V., Boykin D.W., Wilson W.D. (2023). X-ray structure characterization of the selective recognition of at base pair sequences. ACS Bio Med. Chem..

[32] Hunter W.N., Brown T., Kneale G., Anand N.N., Rabinovich D., Kennard O. (1987). The structure of guanosine-thymidine mismatches in B-DNA at 2.5-A resolution. J. Biol. Chem..

[33] Shui X.Q., McFail-Isom L., Hu G.G., Williams L.D. (1998). The B-DNA dodecamer at high resolution reveals a spine of water on sodium. Biochemistry.

[34] Abraham M.J., Murtola T., Schulz R., Páll S., Smith J.C., Hess B., Lindahl E. (2015). GROMACS: High performance molecular simulations through multi-level parallelism from laptops to supercomputers. SoftwareX.

[35] Huang J., Rauscher S., Nawrocki G., Ran T., Feig M., de Groot B.L., Grubmuller H., MacKerell A.D. (2017). CHARMM36m: An improved force field for folded and intrinsically disordered proteins. Nat. Methods.

[36] Li Z., Chen S., Gao C., Yang Z., Shih K.C., Kochovski Z., Yang G., Gou L., Nieh M.P., Jiang M. (2019). Chemically controlled helical polymorphism in protein tubes by selective modulation of supramolecular interactions. J. Am. Chem. Soc..

[37] Lee M., Kim S.H., Hong S.C. (2010). Minute negative superhelicity is sufficient to induce the B-Z transition in the presence of low tension. Proc. Natl. Acad. Sci. USA.

[38] Shin S.-I., Ham S., Park J., Seo S.H., Lim C.H., Jeon H., Huh J., Roh T.-Y. (2016). Z-DNA-forming sites identified by ChIP-Seq are associated with actively transcribed regions in the human genome. DNA Res..

[39] Xia J., Yang L., Dong L., Niu M., Zhang S., Yang Z., Wumaier G., Li Y., Wei X., Gong Y. (2018). Cefminox, a Dual Agonist of Prostacyclin Receptor and Peroxisome Proliferator-Activated Receptor-Gamma Identified by Virtual Screening, Has Therapeutic Efficacy against Hypoxia-Induced Pulmonary Hypertension in Rats. Front. Pharmacol..

[40] Yang Z., Zhao Y., Hao D., Ren S., Yuan X., Meng L., Zhang S. (2020). Bindings of PPARgamma ligand-binding domain with 5-cholesten-3beta, 25-diol, 3-sulfate: Accurate prediction by molecular simulation. J. Biomol. Struct. Dyn..

[41] Berendsen H.J.C., Postma J.P.M., Vangunsteren W.F., Dinola A., Haak J.R. (1984). Molecular-Dynamics with Coupling to an External Bath. J. Chem. Phys..

[42] Darden T., York D., Pedersen L. (1993). Particle mesh Ewald: An N [center-dot] log(N) method for Ewald sums in large systems. J. Chem. Phys..

[43] Hess B., Bekker H., Berendsen H.J.C., Fraaije J.G.E.M. (1997). LINCS: A linear constraint solver for molecular simulations. J. Comput. Chem..

