# Supplementary material for: Exploration of the Character Representation of DNA Chiral Conformations and Deformations via a Curved Surface Discrete Frenet Frame
Source: Int J Mol Sci. 2023 Dec 19;25(1):4. doi: 10.3390/ijms25010004 (PMC10778681; doi:10.3390/ijms25010004)
Supplement: Supplementary file 1 [file ijms-25-00004-s001.zip › ijms-2756681-supplementary.pdf]

Supplementary information

Exploration of the Character Representation of DNA Chiral Conformations and Deformations via a Curved Surface Discrete Frenet Frame

Ying Wang [1], He Wang [1], Shengli Zhang [1], Zhiwei Yang [1], Xuguang Shi [2,*] and Lei Zhang [1,*]

1. Figures and Table of bare DNA parameters.

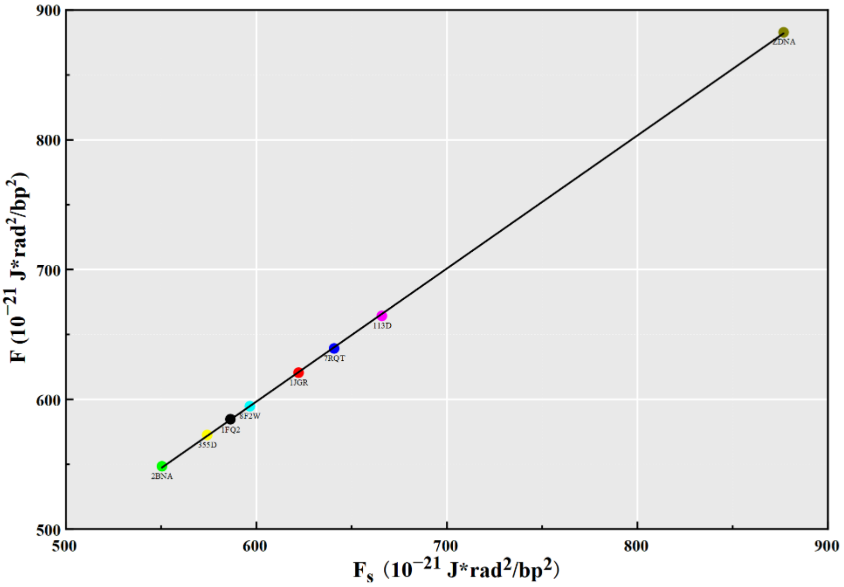

Figure S1. Boxplots of F and F_s for different PDBs. The black line is the linear fit to the data points. In addition, it can be seen from the numerical results that F_s contributes a lot to the overall free energy and accounts for the main part.

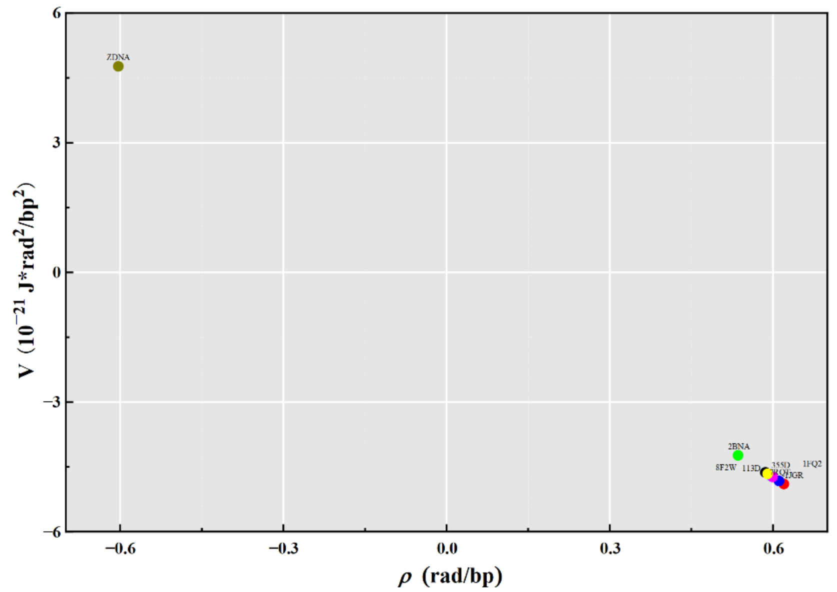

Figure S2. Boxplots of V and ρ for different PDBs. The reason for the seemingly linear graph is that effective potential is an independent quantity, which seems to be only related to the interaction between base pairs inside DNA.

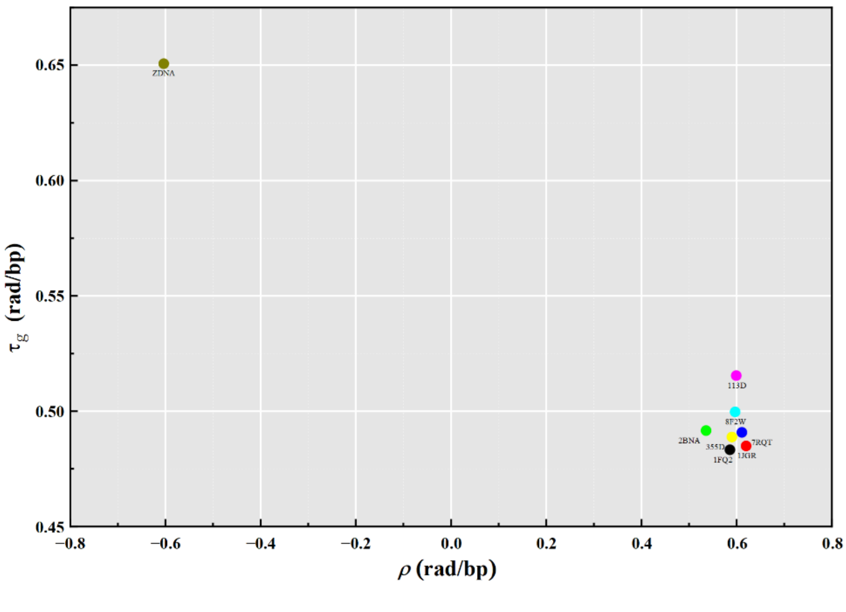

Figure S3. Boxplots of τ_g and ρ for different PDBs.

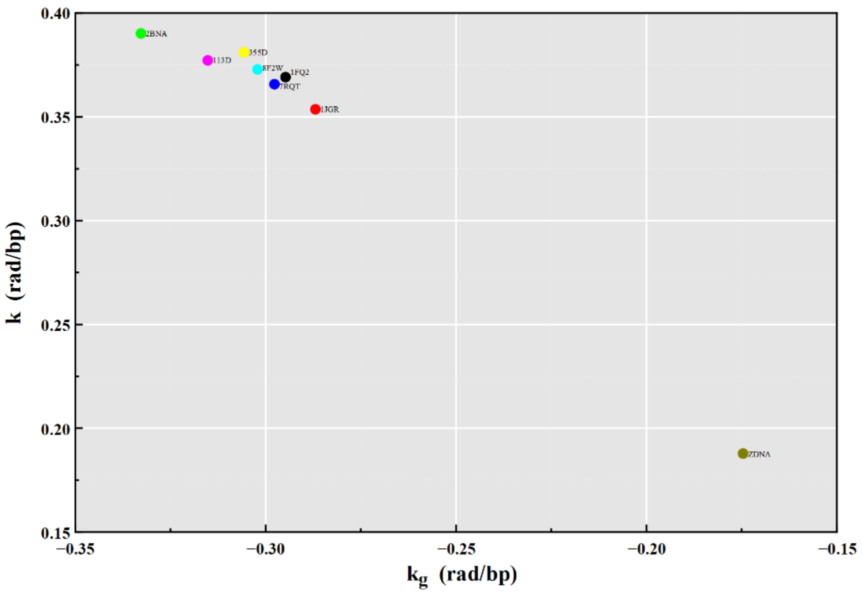

Figure S4. Boxplots of k and k_g for different PDBs. Geodesic curvature contributes significantly to the overall curvature.

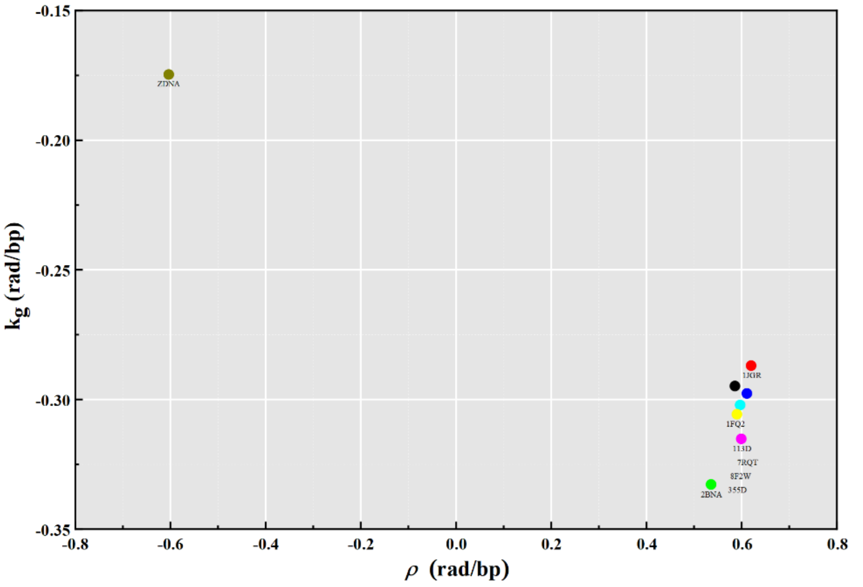

Figure S5. Boxplots of k_g and ρ for different PDBs. Values and properties of geodesic curvature is similar to curvature.

The average geometric parameters and free energy density etc. of the different encoded DNAs are recorded. The values of the parameters are listed in **Table S1**

Table S1. Bare DNA parameters.

Data base	$\bar{k}_g$ [a]	$\bar{k}_n$ [b]	$\bar{k}$ [c]	$\bar{\tau}_g$ [d]	$\frac{d\bar{\chi}}{ds}$ [e]	$\bar{\rho}$ [f]	$\bar{\tau}$ [g]	$\bar{V}$ [h]	$\bar{F}_s$ [i]	$\bar{F}$ [j]
1FQ2[1]	**−0.295**	**−0.023**	**0.369**	**0.483**	**0.169**	**0.586**	**0.066**	**−4.628**	**586.329**	**584.668**
1JGR [2]	**−0.287**	**−0.030**	**0.354**	**0.485**	**0.175**	**0.620**	**0.040**	**−4.897**	**620.540**	**620.540**
2BNA[3]	**−0.333**	**0.004**	**0.390**	**0.492**	**0.147**	**0.536**	**0.103**	**−4.233**	**548.385**	**548.385**
7RQT [4]	**−0.298**	**−0.028**	**0.366**	**0.491**	**0.174**	**0.611**	**0.054**	**−4.827**	**639.228**	**639.228**
8F2W[5]	**−0.302**	**−0.026**	**0.373**	**0.500**	**0.168**	**0.597**	**0.071**	**−4.715**	**594.680**	**594.680**
113D[6]	**−0.315**	**−0.035**	**0.377**	**0.515**	**0.152**	**0.599**	**0.068**	**−4.733**	**664.295**	**664.295**
355D[7]	**−0.306**	**−0.025**	**0.381**	**0.489**	**0.171**	**0.590**	**0.070**	**−4.662**	**572.524**	**572.524**
Z-DNA	**−0.175**	**−0.010**	**0.188**	**0.651**	**−0.162**	**−0.604**	**1.092**	**4.768**	**882.897**	**882.897**

[a] k_g represents the axial geodesic curvature of the spiral, [b] k_n is the normal curvature, [c] $k=\sqrt{k_n^2+k_g^2}$ is the global curvature of the spiral axis, [d] τ_g is the geodesic torsion, [e] $\frac{d\chi}{ds}$ is the rate of change of the initial angle, [f] ρ is the base pair torsion angle per unit length, [g] $\tau=\sqrt{(\tau_g+\frac{d\chi}{ds}-\rho)^2}$ is the global twist of the spiral axis, [h] $V=\tau_c\rho$ is the potential energy density, [i] F_s is the structural free energy density, and [j] F is the overall free energy density.

2. Molecular dynamics (MD) simulations.

The structure of Z-DNA was conducted by homology model method (Accelrys. Discovery Studio 3.1. Available online: http://accelrys.com (accessed on 8 November 2022)) and were further equilibrated by MD simulations, using using GROMACS 2018.8 [8] and Charmm36m force field [9] Details of the MD simulation setup are in agreement with our previous works [10-12]. In brief, each system was solvated in a cubic box of SPC/E (simple-point-charge) water molecules extending at least 10.0 Å from any solute atom. Na+ counter-anions were placed to neutralize the system. To mimic physiological conditions, the NPT ensemble was applied at constant pressure (1 atm) and 300 K [13]. Particle-mesh Ewald (PME) method [14] and LINCS algorithm [15] were applied to handle long-range electrostatics and constrain all covalent bonds. The cutoff radii for coulomb and van der Waals interactions were set to 9.0 Å. Free dynamics were performed using a 2.0 fs time step, and coordinates were collected every 10.0 ps.

After MD optimization, we obtained the DNA structure diagram Figure 6. Considering that the bases at the head and tail of the DNA may be open due to environmental influences, we selected the middle segment of the result as a cycle segment to study in the text.

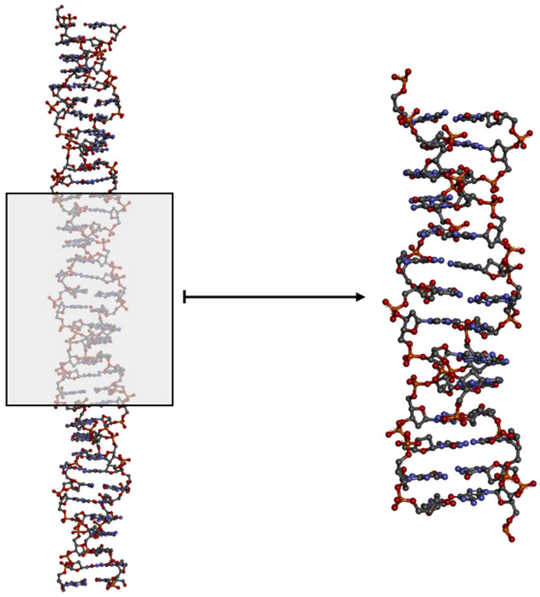

Figure 6. MD result of Z-DNA.

References

1. Sines, C. C.; McFail-Isom, L.; Howerton, S. B.; VanDerveer, D.; Williams, L. D., Cations mediate B-DNA conformational heterogeneity. *Journal of the American Chemical Society* **2000,** 122, 11048-11056.
2. Howerton, S. B.; Sines, C. C.; VanDerveer, D.; Williams, L. D., Locating monovalent cations in the grooves of B-DNA. *Biochemistry-Us* **2001,** 40, 10023-10031.
3. Drew, H. R.; Samson, S.; Dickerson, R. E., STRUCTURE OF A B-DNA DODECAMER AT 16-K. *P Natl Acad Sci-Biol* **1982,** 79, 4040-4044.
4. Chen, C.; Fang, Z.; Huang, Z., 2 '-beta-Selenium Atom on Thymidine to Control beta-Form DNA Conformation and Large Crystal Formation. *Crystal Growth & Design* **2022,** 22,, 3601-3604.
5. Edwin N. Ogbonna, A. P., Abdelbasset A. Farahat, J. Ross Terrell, Ekaterina Mineva, Victor Ogbonna, David W Boykin, and W. David Wilson, X-ray Structure Characterization of the Selective Recognition of AT Base Pair Sequences. *ACS Bio Med Chem* **2023**.
6. Hunter, W. N.; Brown, T.; Kneale, G.; Anand, N. N.; Rabinovich, D.; Kennard, O., THE STRUCTURE OF GUANOSINE-THYMIDINE MISMATCHES IN B-DNA AT 2.5-A RESOLUTION. *Journal of Biological Chemistry* **1987,** 262, 9962-9970.
7. Shui, X. Q.; McFail-Isom, L.; Hu, G. G.; Williams, L. D., The B-DNA dodecamer at high resolution reveals a spine of water on sodium. *Biochemistry-Us* **1998,** 37, 8341-8355.
8. Abraham, M. J.; Murtola, T.; Schulz, R.; Páll, S.; Smith, J. C.; Hess, B.; Lindahl, E., GROMACS: High performance molecular simulations through multi-level parallelism from laptops to supercomputers. *Softwarex* **2015,** 1-2, 19-25.
9. Huang, J.; Rauscher, S.; Nawrocki, G.; Ran, T.; Feig, M.; de Groot, B. L.; Grubmuller, H.; MacKerell, A. D., CHARMM36m: an improved force field for folded and intrinsically disordered proteins. *Nature methods* **2017,** 14, 71-73.
10. Xia, J.; Yang, L.; Dong, L.; Niu, M.; Zhang, S.; Yang, Z.; Wumaier, G.; Li, Y.; Wei, X.; Gong, Y.; Zhu, N.; Li, S., Cefminox, a Dual Agonist of Prostacyclin Receptor and Peroxisome Proliferator-Activated Receptor-Gamma Identified by Virtual Screening, Has Therapeutic Efficacy against Hypoxia-Induced Pulmonary Hypertension in Rats. *Front Pharmacol* **2018,** 9, 134.
11. Yang, Z.; Zhao, Y.; Hao, D.; Ren, S.; Yuan, X.; Meng, L.; Zhang, S., Bindings of PPARgamma ligand-binding domain with 5-cholesten-3beta, 25-diol, 3-sulfate: accurate prediction by molecular simulation. *J Biomol Struct Dyn* **2020,** 38,, 1918-1926.
12. Li, Z.; Chen, S.; Gao, C.; Yang, Z.; Shih, K. C.; Kochovski, Z.; Yang, G.; Gou, L.; Nieh, M. P.; Jiang, M.; Zhang, L.; Chen, G., Chemically Controlled Helical Polymorphism in Protein Tubes by Selective Modulation of Supramolecular Interactions. *J Am Chem Soc* **2019,** 141, 19448-19457.
13. Berendsen, H. J. C.; Postma, J. P. M.; Vangunsteren, W. F.; Dinola, A.; Haak, J. R., Molecular-Dynamics with Coupling to an External Bath. *Journal of Chemical Physics* **1984,** 81, 3684-3690.
14. Darden, T.; York, D.; Pedersen, L., Particle mesh Ewald: An N [center-dot] log(N) method for Ewald sums in large systems. *J Chem Phys* **1993,** 98, 10089-10092.
15. Hess, B.; Bekker, H.; Berendsen, H. J. C.; Fraaije, J. G. E. M., LINCS: A linear constraint solver for molecular simulations. *Journal of Computational Chemistry* **1997,** 18, 1463-1472.
